# TCM Regulates PI3K/Akt Signal Pathway to Intervene Atherosclerotic Cardiovascular Disease

**DOI:** 10.1155/2021/4854755

**Published:** 2021-12-16

**Authors:** Jiali Liu, Pangao Xu, Dekun Liu, Ruiqing Wang, Shengnan Cui, Qiuyan Zhang, Yunlun Li, Wenqing Yang, Dan Zhang

**Affiliations:** ^1^Faculty of Traditional Chinese Medicine, Shandong University of Traditional Chinese Medicine, Jinan, Shandong, China; ^2^First Clinical School of Medicine, Shandong University of Traditional Chinese Medicine Shandong, Jinan, Shandong, China; ^3^Innovation Research Institute of Traditional Chinese Medicine, Shandong University of Traditional Chinese Medicine, Jinan, Shandong, China; ^4^Pharmacy School, Shandong University of Traditional Chinese Medicine Shandong, Jinan, Shandong, China; ^5^Affiliated Hospital of Shandong University of Traditional Chinese Medicine, Jinan, Shandong, China; ^6^Shandong Engineering Research Center of Traditional Chinese Medicine Precise Treatment of Cardiovascular Disease, Zibo, Shandong, China; ^7^Experimental Center, Shandong University of Traditional Chinese Medicine, Jinan, Shandong, China

## Abstract

Vascular endothelial injury is the initial stage of atherosclerosis (AS). Stimulating and activating the phosphoinositide 3-kinase (PI3K)/protein kinase B (Akt) signaling pathway can regulate the expression of vascular endothelial cytokines, thus affecting the occurrence and development of AS. In addition, the PI3K/Akt signaling pathway can regulate the polarization and survival of macrophages and the expression of inflammatory factors and platelet function, thus influencing the progression of AS. In recent years, traditional Chinese medicine (TCM) has been widely recognized for its advantages of fewer side effects, multiple pathways, and multiple targets. Also, the research of TCM regulation of AS via the PI3K/Akt signaling pathway has achieved certain results. This study aimed to analyze the characteristics of the PI3K/Akt signaling pathway and its role in the pathogenesis of AS, as well as the role of Chinese medicine in regulating the PI3K/Akt signaling pathway. The findings are expected to provide a theoretical basis for the clinical treatment and pathological mechanism research of AS.

## 1. Introduction

The incidence and mortality of cardiovascular diseases continue to increase with the development of society and economy, the aging of the population, and the acceleration of urbanization. At present, the mortality rate of cardiovascular diseases in China is 46.66% in rural areas and 43.81% in urban areas; hence, these diseases are the first cause of death among urban and rural residents [1]. Atherosclerotic cardiovascular disease (ASCVD), represented by coronary heart disease and stroke, has become one of the main causes of death [2]. The pathological basis of ASCVD is atherosclerosis (AS), which is a cardiovascular disease induced by multiple risk factors. AS is caused by the dysfunction of vascular smooth muscle cells and endothelial cells and pro-inflammatory cytokines produced by activated macrophages [3]. Recent studies have found that the PI3K/Akt signaling pathway plays a vital role in the occurrence and development of AS. After PI3K activates Akt through phosphorylation, it can effectively act on the downstream target proteins B-cell lymphoma-2 gene-associated promoter (Bad), cysteine-aspartic protease (caspase-9), glycogen synthase kinase-3*β* (GSK-3*β*), mammalian target of rapamycin (mTOR), and endothelial nitric oxide synthase (eNOS) and play an important role in cell proliferation, apoptosis, autophagy, and so forth [4,5]. Activating the PI3K/Akt signaling pathway can reduce reactive oxygen species (ROS) and lipid deposition levels, inhibit plaque formation, and reverse the progression of AS [6]. At the same time, the PI3K/Akt signaling pathway plays a key role in regulating the survival, proliferation, and migration of macrophages, as well as cell metabolism and the secretion and release of inflammatory factors.

At present, the drugs used in western medicine to treat AS mainly include anti-platelet aggregation drugs, statins, lipid-lowering drugs, *β*-receptor blockers, vasoactive drugs, cyclooxygenase inhibitors, anti-lipid oxidants, and vascular tension-lowering drugs [7]. These drugs have obvious side effects, which can cause liver and kidney function damage. In contrast, traditional Chinese medicine (TCM) is characterized by the holism concept, as well as syndrome differentiation and treatment. It has gradually attracted wide attention and application because of its obvious effects, stable efficacy, few side effects, safety, and cheapness. It has shown that TCM can restrain the occurrence of cardiovascular diseases including AS through multi-target pathways such as anti-inflammatory, anti-oxidative stress, and regulation of metabolism based on the PI3K/Akt signaling pathway. Therefore, this study aimed to summarize and analyze the mechanism of the PI3K/Akt signaling pathway in AS and the regulatory role played by TCM through this pathway. The findings are expected to provide strategic directions for the pathological mechanism and treatment of AS.

## 2. The Molecular Regulation Mechanism of PI3K/Akt Signaling Pathway

As an intracellular signal transduction pathway, PI3K/Akt pathway responds to extracellular signals and plays a crucial role in regulating various cell and molecular functions, such as metabolism, proliferation, cell survival, growth, and angiogenesis. This process is mediated by serine or threonine phosphorylation of a range of downstream substrates, in which PI3K and Akt are the key genes.

### 2.1. The Structure and Mechanism of PI3K

PI3K has serine/threonine (Ser/Thr) kinase activity as well as phosphatidylinositol kinase activity; it is widely present in various types of cells. It can be activated by the toll-like receptor (TLR) 4 and other pathogen recognition receptors, cytokines, chemokines, and Fc receptors [8,9]. PI3K can be divided into type I, type II, and type III according to the structure, regulation mode, and substrate specificity. Type I PI3K is a heterodimer composed of a cleavage subunit (p110*α*, p110*β*, p110*δ*, and p110*γ*) and a regulatory subunit (p85 or p101 family). It is the only heterodimer that phosphorylates the peptide (4, 5) p2 into a peptide (3, 4, 5) P3 [10]; PI3K*γ* is expressed in cardiovascular tissues such as the heart and vascular system [8]. In the PI3K family, the most widely studied is a type I PI3K that can be activated by cell surface receptors; type II and III PI3K are less studied. Class I PI3K in mammalian cells is divided into two subtypes, IA and IB, which transmit signals from tyrosine kinase-linked receptors and G protein-linked receptors, respectively [11]. Type II PI3K has only a catalytic subunit; the lack of an aspartic acid residue at the carboxyl end makes it difficult for Ca^2+^ to bind to it. Therefore, type II PI3K does not depend on intracellular Ca^2+^ for its function. Type III PI3K is similar in structure to type I, consisting of catalytic subunits and regulatory subunits; it only catalyzes PI to produce 3,4,5-phosphatidylinositol (PIP3) and participates in the delivery of proteins and vesicles [9,12]. PI3K can be activated in two ways. One is to interact with growth factor receptors or connexins that have phosphorylated tyrosine residues, causing the dimer to change conformation and be activated; the other is to activate PI3K through the direct binding of small GTPases protein superfamily Ras and p110. The result of PI3K activation is the production of the second messenger PIP3 on the plasma membrane. PIP3 binds to the signal protein Akt and phosphoinositide-dependent kinase 1 (PDK1) containing the pleckstrin homology domain in the cell, prompting PDK1 to phosphorylate the Ser308 site of the Akt protein and leading to the activation of Akt [13].

### 2.2. The Structure and Mechanism of Akt

Akt, also known as protein kinase B (PKB), is a serine/threonine kinase and a downstream factor of PI3K. Akt binds to PIP3 on the membrane to allow these proteins to approach and promote the phosphorylation of Akt by PDK1 [13]. In addition, Akt can also be activated via the phosphorylation of Thr473 by 3-phosphoinositide-dependent protein kinase 2 (PDK2). The activation of Akt can inhibit the phosphorylation of apoptosis signaling proteins or induce transcription factors to regulate cell apoptosis. Activated Akt can also activate or inhibit its downstream target proteins Bad, caspase-9, GSK-3*β*, mTOR, nuclear transcription factor-*κ*B (NF-*κ*B), and eNOS through phosphorylation, thereby regulating cell proliferation, differentiation, and migration [4]. At the same time, the activity level of the Akt signal determines the viability of monocytes/macrophages in AS lesions and their resistance to pro-apoptotic stimuli [14]. Currently, three different Akt subtypes are known: Akt1, Akt2, and Akt3. They have significant homologous sequences, similar structures, and different biological functions. Akt1 is an enzyme mainly related to cardiovascular function, which can regulate the production of eNOS and NO. The absence of Akt1 increases the perinatal mortality rate of mice [15–17]. Moreover, inhibiting Akt1 in macrophages promotes the polarization of M1 and the formation of AS [9,18]. Similarly, mice lacking Akt2 are prone to hyperglycemia and insulin resistance; the absence of Akt2 in macrophages promotes the anti-inflammatory M2 phenotype and reduces AS [19]. Mice lacking Akt3 affect their glucose metabolism, body weight, and abnormal brain cell development [19].

## 3. The Role of PI3K/Akt Signaling Pathway in AS

AS is a lesion with functional impairment under various cardiovascular risk factors, accompanied by inflammatory cell infiltration, altered permeability of vascular endothelial cells, lipid deposition into the lower intima, thickening and hardening arterial wall, and vascular cavity stenosis [20–22]. The pathological process from atherosclerotic plaque formation to plaque rupture involves extremely complex cell signaling pathways. The PI3K/Akt signaling pathway is an important pathway in the pathogenesis of AS. It has been found that among the different PI3K isoforms, PI3K*γ* is highly expressed in the haematopoietic cell lineage and hence dominates the inflammation of AS [8]. PI3K*γ* can be activated by several chemokines, pro-inflammatory lipids, and vasoactive stimuli, downstream of Gi-coupled receptors. Moreover, ox-low-density lipoprotein (LDL) and angiotensin II (Ang II) activate PI3K in macrophages, which can increase the activity of macrophages and enhance the immune-inflammatory response [23]. Furthermore, activation of PI3K/Akt signaling is also shown downstream of other related atherogenic stimuli including interferon *γ*, transforming growth factor *β*, and TNF-a, which connect to different receptor types [8]. Indeed, the activation of the PI3K/Akt pathway can inhibit vascular smooth muscle cells migration, reduce platelet adhesion, inhibit the expression of inflammatory factors, reduce vascular endothelial cell apoptosis, and repair vascular endothelial damage [24]. It indicates that the development of AS can be affected by PI3K/Akt signaling pathway.

### 3.1. PI3K/Akt Signaling Pathway Repairs Vascular Endothelium and Antagonizes AS

**Figure 1:**
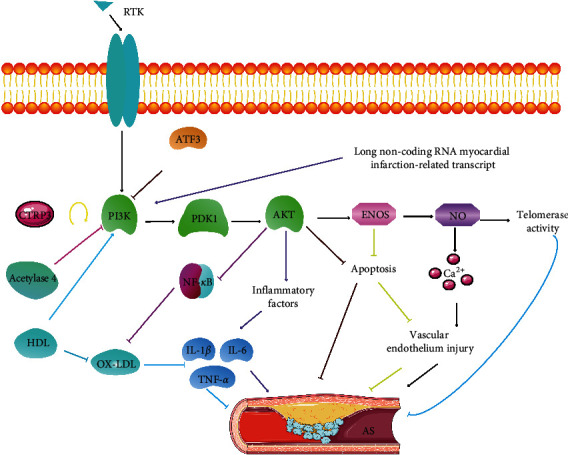
PI3K/Akt signaling pathway activates inflammation and repairs vascular endothelium and participates in the development of AS. ↑ represents activation, and ⊥ represents inhibition.

The endothelial-dependent vascular dysfunction is a precursor to the formation of AS; one of the main features of endothelial dysfunction is the reduced biological activity of NO derived from eNOS. NO is mainly produced by endothelial cells under the catalysis of the important downstream gene eNOS of the PI3K/Akt signaling pathway. Akt is activated by PI3K to promote the synthesis and release of endogenous NO through the phosphorylation of eNOS, which protects the vascular endothelium [25,26]. When the NO concentration and activity level in the body decrease, the free Ca^2+^ concentration in the cells increases, which causes the constriction of blood vessels. At the same time, it changes the cell permeability, makes the vascular endothelial function abnormal, and accelerates the AS process [27]. Chen et al. [28] found that C1q/tumor necrosis factor-related protein-3 (CTRP3) could significantly reverse the oxidized ox-LDL-induced apoptosis, improve the inhibition of PI3K/Akt/eNOS phosphorylation, and be involved in endothelial repair during the AS development. It is suggested that the protective effect of CTRP3 against ox-LDL-induced inflammation and endothelial dysfunction can be mediated by regulating the PI3K/Akt/eNOS pathway during the AS pathogenesis [28]. High-density lipoprotein (HDL) promotes the reverse transport of cholesterol, inhibits the oxidation of LDL, and reduces the levels of inflammatory cytokines and vascular leukocyte adhesion molecules [29]. It promotes endothelial progenitor-mediated endothelial repair capabilities by increasing the number and function of endothelial progenitors [26]. In addition, HDL achieves anti-ASCVD action by promoting telomerase activity through increasing the NO level and promoting the PI3K/Akt signaling pathway to delay endothelial progenitor cell aging [26]. Thus, it shows that repairing vascular endothelium through the PI3K/Akt signaling pathway has a potential application value in treating cardiovascular diseases (Figure 1).

### 3.2. PI3K/Akt Signaling Pathway Regulates Cell Metabolism and Affects AS

**Figure 2:**
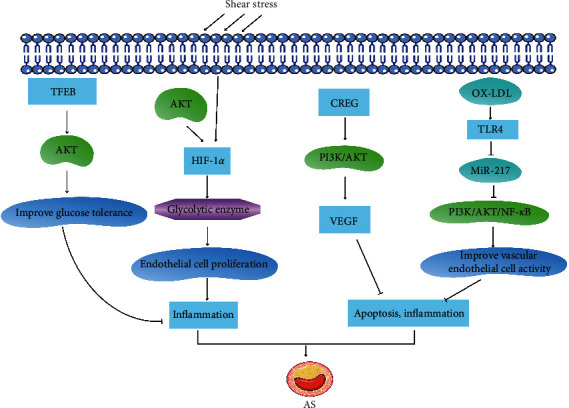
PI3K/Akt signaling pathway regulates cell metabolism and affects AS. ↑ represents activation, and ⊥ represents inhibition.

Endothelial cell metabolism plays an important role in health maintenance and disease occurrence. Its main metabolic pathways include glycolysis, fatty acid oxidation, and amino acid metabolism. It is used in a variety of vascular-related diseases, such as AS, diabetes, and retinal neovascular disease. Tumors are accompanied by varying degrees of endothelial cell metabolism disorders [30]. For example, endothelial cell glycolysis disorder can cause AS. When Akt is activated, it promotes phosphorylation of the downstream target protein GSK-3 and increases the expression of HIF-1*α* [31]. In addition, Akt also promotes the transcription of HIF-1*α* by activating the expression of mTOR [32]. Shear stress can change endothelial cell metabolism by activating hypoxia-inducible factor 1a (HIF-1a). Furthermore, HIF-1a induces endothelial cell proliferation and inflammation by activating glycolytic enzymes and promotes the occurrence of AS [33]. Transcription factor EB (TFEB) is the main regulator of lysosomal biogenesis and autophagy and has a protective effect on vascular inflammation and AS. Sun et al. [34] found that the endothelial TFEB could enhance the Akt signal in endothelial cells and metabolically active tissues and improve glucose tolerance *in vivo*. Abnormal endothelial cell metabolism causes vascular dysfunction; during endothelial damage, apoptosis starts, causing vascular leakage, inflammation, and coagulation [35]. Studies have found that ox-LDL can induce the expression of TLR4 in endothelial cells and inhibit miR-217 and PI3K/Akt/NF-*κ*B pathways. MiR-217 can increase the activity of AS vascular endothelial cells through the TLR4/PI3K/Akt/NF-*κ*B signal transduction pathway and inhibit their apoptosis, inflammation, and endothelial-mesenchymal transition [36]. Long noncoding RNA myocardial infarction-related transcripts can promote the aortic cell apoptosis in mice with AS by activating the PI3K/Akt signaling pathway [37]. E1A stimulating gene cytostatic (CREG) is a homeostasis regulatory gene abundantly expressed in adult arterial endothelium. CREG protects human umbilical vein endothelial cells (HUVECs) from apoptosis and inhibits AS endothelial cell apoptosis by activating its downstream PI3K/Akt signaling pathway and increasing the secretion of vascular endothelial growth factor [24]. Activating the PI3K/Akt signaling pathway to inhibit endothelial cell apoptosis is the key to treating AS, and anti-apoptotic treatments may be used to treat AS (Figure 2).

### 3.3. PI3K/Akt Signaling Pathway Regulates Platelets and Affects AS

**Figure 3:**
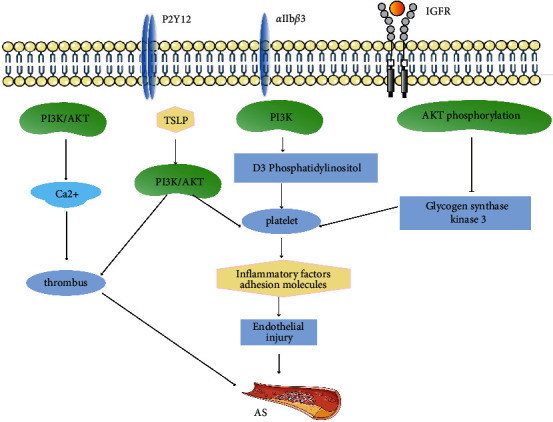
PI3K/Akt signaling pathway regulates platelets and affects AS. ↑ represents activation, and ⊥ represents inhibition.

Under normal physiological conditions, platelets participate in the coagulation process of the human body. When the blood vessels of the body are damaged, the collagen exposed by vascular endothelial cells activates the coagulation factors of the human body, which in turn causes platelet activation, leading to an increase in the platelet aggregation rate [38]. Platelet activation can produce platelet microparticles. These microparticles adhere to the blood vessel wall to release arachidonic acid, induce inflammatory factors and adhesion molecules to attach to endothelial cells, reduce NO synthesis and NOS activity, damage endothelial function, and accelerate the progression of AS. The acute complications of AS plaques (e.g., plaque rupture) can cause platelet aggregation and serious clinical events such as myocardial infarction and stroke [8]. PI3K and its downstream effector Akt play a decisive role in regulating platelet function [39]. PI3K synthesizes D3 phosphatidylinositol and regulates many important platelet reactions, such as platelet shape changes, integrin *α*IIb*β*3 activation, and irreversible platelet aggregation [40]. At the same time, Akt phosphorylation can restrain glycogen synthase kinase-3, promoting blood clot contraction, platelet deformation and expansion, and an increase in thrombus volume [41]. Studies have found that PI3K is activated downstream of several membrane proteins in platelets, including G protein-coupled receptors (such as P2Y12), tyrosine kinases (such as IGFR), *α*IIb*β*3, and so forth, which are widely involved in regulating adhesion and aggregation [8]. On the contrary, when PI3K/Akt enhances calcium release and *α*IIb*β*3 activation, it can promote thrombosis [8]. Thymic stromal lymphopoietin (TSLP) is a new type of interleukin 7 (IL-7) cytokine that plays an important role in inflammatory diseases and is overexpressed in human AS arterial specimens [42]. Dong et al. [43] found that the surface of mouse platelets had functional TSLP receptor expression. TSLP triggers platelet activation and thrombosis by relying on the PI3K/Akt signaling pathway, suggesting an important role of TSLP in vascular inflammation and thrombo-occlusive diseases. Hence, the regulation of the PI3K/Akt signaling pathway may become a new potential method of antithrombotic therapy (Figure 3).

### 3.4. PI3K/Akt Signaling Pathway Activates Inflammation and Participates in the Occurrence and Development of AS

Vascular inflammation plays an important role in the occurrence and development of AS. After a vascular endothelial injury, endothelial cells are activated and produce inflammatory factors. These substances attract monocytes and neutrophils, which attach to the activated endothelial cells, penetrate the artery wall, and cause inflammation. The activated PI3K/Akt signaling pathway can promote the accumulation of inflammatory cells to accelerate the development of AS. Long noncoding RNA myocardial infarction-related transcripts can promote the expression of inflammatory factors (IL-1*β*, IL-6, and TNF-*α*) in mice with AS activating the PI3K/Akt signaling pathway and aggravate AS damage [37]. The inhibition of the PI3K/Akt signaling pathway can significantly reduce serum-free fatty acid, cholesterol, and triglyceride levels in mice and inhibit the lipopolysaccharide (LPS)-induced secretion of pro-inflammatory mediators in monocytes/macrophages [44]. Inhibiting the PI3K/Akt signaling pathway can inhibit the expression of NF-*κ*B to improve the progression of AS [45]. Acetylase 4 inhibits the expression of inflammatory cytokines in HUVECs induced by ox-LDL by inhibiting the PI3K/Akt/NF-*κ*B signaling pathway [46]. On the contrary, naringin can reduce the damage to HUVECs caused by oxidative stress and inflammation by activating the PI3K/Akt pathway and inhibiting the NF-*κ*B pathway, preventing endothelial dysfunction, and improving the development of AS. Activating transcription factor 3 (ATF3) inhibits the inflammatory response by regulating the expression of cytokines and chemokines and mediates the progression and stability of AS plaques [21]. At the same time, ATF3 can stabilize the progression of AS plaques by inhibiting the PI3K/Akt pathway and inhibiting the aggregation and apoptosis of macrophages [21]. The aforementioned findings indicate that PI3K/Akt can affect the occurrence and development of AS through inflammatory factors or pro-inflammatory mediators (Figure 1).

## 4. TCM Treatment Strategy of PI3K/Akt Signaling Pathway to Prevent and Treat AS

TCM has the characteristics of overall regulation, syndrome differentiation, and treatment and has unique advantages in anti-AS. The effective ingredients of many classic compounds and Chinese medicine monomers have a good effect on the PI3K/Akt signaling pathway and can effectively reduce AS.

### 4.1. TCM and Its Extract Prevent as through PI3K/Akt Signaling Pathway

The atherosclerotic lesion process is a chronic inflammatory process, while a great number of TCM has an inhibitory effect on the inflammatory response. Macrophages produce inflammatory cytokines and lipid mediators and participate in the plaque inflammation response [47]. Tanshinone IIa (TS IIA) is the main fat-soluble active ingredient of Danshen. Baicalin IV (AS IV) is a cycloartane-type triterpene glycoside isolated from astragalus. Wang et al. [48] found that TS IIA and AS IV enhanced vascular stability by activating PI3K/Akt and inhibiting TLR4/NF-*κ*B signaling. The PI3K/Akt signaling pathway downregulates TLR4/NF-*κ*B signaling, reduces inflammation and the expression of matrix metalloproteinase-9 (MMP-9), and suppresses unstable plaques, thereby inhibiting the occurrence and development of AS. Quercetin is a dietary flavonoid compound extracted from various plants (apples and onions). It has a wide range of pharmacological properties, such as anti-inflammatory and anti-oxidant properties [49,50]. Quercetin can improve the progression of AS induced by high fructose feeding via enhancing PI3K/Akt and inhibiting ROS, thereby alleviating apoptosis and inflammation [51]. In addition, quercetin can interfere with the activity of key proteins in the PI3K/Akt pathway, suppress NF-*κ*B transfer, inhibit the proliferation and secretion of aortic wall fibroblasts and smooth muscle cells, and delay the process of AS [52]. Emodin is the main component of the free anthraquinone of *Polygonum multiflorum*. It can exert an anti-atherosclerotic effect through its lipid-lowering and anti-inflammatory activities. Studies have found that *Polygonum* emodin can improve dyslipidemia in ApoE^–/–^ mice, inhibit the activity of PI3K/Akt and its downstream mTOR signaling pathway, stimulate the increase in autophagy, increase the metabolism of glycolipids, and achieve the anti-AS effect [53].

Oxidative stress in blood vessels is the main risk factor for vascular endothelial cell damage and AS [54]. Breviscapine is an extract of wild baicalin, which promotes blood circulation, removes blood stasis, lowers blood lipids, scavenges oxygen free radicals, and exerts antioxidant effects [55]. Fan Hua [55] found that scutellarin activated the PI3K/Akt signaling pathway and upregulated PIP3. Furthermore, PIP3 bound to the signaling protein Akt and underwent a conformational change to phosphorylate and activate Akt. Activated Akt played a role in regulating the downstream factor nuclear factor E2-related factor 2 (Nrf2), so that phosphorylated Nrf2 increased nuclear translocation ability and then exerted its protective effects such as anti-oxidative stress, thereby regulating AS. Salidroside (SAL) is a phenylpropionoside isolated from the medicinal plant *Rhodiola*. Studies have found that increasing the level of oxidative stress can lead to lipid peroxidation damage and promote the formation and development of AS. The inhibition of PI3K/Akt/mTOR signaling pathway inhibits the expression of HIF-1*α*; under oxidative stress, the antioxidant effect of SAL depends on the activation of HIF-1*α* [32]. SAL can significantly reduce the mitochondrial membrane potential to reduce ATP production, thereby increasing the cellular ratio of adenosine monophosphate (AMP) to ATP, and then activate AMP-dependent protein kinase (AMPK) [16]. Activated AMPK directly increases the activity of eNOS, leading to the production of NO and improving endothelial function. AMPK also induces the phosphorylation and expression of eNOS through the PI3K/Akt pathway, which has a negative feedback effect on the activation of AMPK [56]. At the same time, the depolarization of the membrane potential reduces the production of superoxide anions (O_2_^–^), increases the bioavailability of NO, improves endothelial function, and ultimately mediates the anti-AS of SAL [16].

Apoptosis is induced by stimuli such as hypoxia and oxidative stress caused by a variety of cardiovascular diseases. It is one of the important factors that promote the development of AS [57]. The root or whole plant of *Gynostemma pentaphyllum* lowers the levels of blood lipids, prevents AS, exerts antioxidant effects, lowers blood sugar, and regulates immunity. *Gynostemma* saponins are the main components of *G. pentaphyllum*, which has a variety of pharmacological activities and is beneficial in treating AS [58]. Gypenoside can effectively regulate the expression of apoptosis-related proteins PI3K/Akt/Bad, enhance the expression of PI3K and p-Akt, and downregulate the expression of p-Bad, Cyt-c, caspase-3, and caspase-9. It suggests that gypenosides can regulate mitochondrial function through the PI3K/Akt/Bad pathway and inhibit the occurrence of AS [59]. Gypenoside XVII (GP-17) is a new type of phytoestrogens belonging to gypenosides, and its structure is similar to that of estradiol. The ROS induced by ox-LDL can lead to endothelial cell apoptosis and oxidative stress. GP-17 can activate the ER*α*-mediated PI3K/Akt pathway, leading to the upregulation of Nrf2/heme oxygenase-1 (HO-1) and the increase in antioxidant enzyme levels, thereby reducing the oxidative damage induced by ox-LDL [60]. At the same time, GP-17 inhibits ox-LDL-mediated HUVEC apoptosis by reducing the ratio of Bax to Bcl-2 and controlling the activation of caspase-3. It suggests that GP-17 inhibits ox-LDL-induced endothelial cell apoptosis through the PI3K/Akt pathway mediated by ER*α* [60]. Isorhamnetin (Iso) is a flavonoid compound extracted from Chinese sea buckthorn, which has anti-inflammatory and anti-oxidant activities. Iso exerts anti-AS effects by activating PI3K/Akt signals and upregulating THP-1-derived macrophage HO-1 in preventing ox-LDL-induced apoptosis and by inhibiting lipid deposition in ox-LDL-induced macrophage apoptosis [6] (Table 1).

### 4.2. Chinese Herbal Compound Prevents AS through PI3K/Akt Signaling Pathway

Abnormal regulation of endothelial cell metabolism can cause a variety of vascular diseases. Danhong injection is a standardized product of TCM extracted from danshen and safflower. The main components are tanshinone, salvianolic acid, and safflower yellow. Danhong injection has anti-inflammatory, anti-thrombotic, lipid-regulatory, and anti-apoptotic effects. It can improve blood glucose and lipid metabolism disorders in mice with high-fat-diet-induced AS [62]. Danhong injection can prevent macrophage lipid accumulation by activating the PI3K/Akt insulin signaling pathway and avoid the formation of macrophage-derived foam cells in a dose-dependent manner, helping improve the prognosis of AS induced by a high-fat diet [62].

The classic TCM prescription Buyang Huanwu decoction and the TCM Qishen capsule for treating AS have the effects of replenishing qi, activating blood, removing blood stasis, and dredging collaterals and can treat qi deficiency and blood stasis type AS [63]. Buyang Huanwu decoction comprises astragalus, red peony root, angelica, chuanxiong, earthworm, peach kernel, and safflower. Studies have found that the medicated serum of Tongbu Yang Huanwu decoction can inhibit the expression of PI3K and p-Akt proteins activated by LPS stimulation and further inhibit downstream inflammatory factors, thereby treating AS and reducing inflammation [64]. Qishen capsule regulates blood lipids; effectively reduces the levels of TG, TC, and LDL-C in the serum of mice; and increases the level of HDL-C. It can inhibit the phosphorylation of PI3K and Akt protein, thereby inhibiting the excessive activation of this signaling pathway and alleviating the AS process in mice [65]. In addition, Huanglian Jiedu decoction also has a good effect of reducing inflammation. Huanglian Jiedu decoction was first published in “Elbow Reserve Emergency Recipe.” It comprises four Chinese medicines, including *Coptis*, *Phellodendron*, *Scutellaria baicalensis* Georgi, and *Gardenia* [66]. Studies have found that Huanglian Jiedu decoction can inhibit the polarization of M1 macrophages, promote the polarization of M2 macrophages, reduce inflammation, and maintain the stability of atherosclerotic plaques in the arteries, thereby exerting an anti-AS effect [67]. Another scholar found that Huanglian Jiedu decoction reduced the expression of AS-related inflammatory factors, improved rabbit AS, reduced AS fibrosis, and increased autophagy [68]. Huanglian Jiedu decoction freeze-dried powder can improve rabbit AS by inhibiting the PI3K/Akt/mTOR pathway to increase autophagy [68].

Endothelial cell proliferation and apoptosis are in a dynamic equilibrium state. When endothelial cells are excessively apoptotic, endothelial dysfunction and endothelial cell damage occur, resulting in the occurrence of AS. Tongxinluo is a compound preparation of TCM for treating cardiovascular and cerebrovascular diseases developed under the guidance of the theory of TCM veins and collaterals. It has the effects of replenishing qi, activating blood, dredging collaterals, and relieving pain and can regulate ASCVD through its anti-inflammatory, anti-oxidant, lipid-regulating, anti-coagulation, and other effects [69]. Liang Junqing et al. found that Tongxinluo upregulated the expression of HIF in vascular endothelial cells through the PI3K/Akt/HIF signaling pathway, promoted anti-apoptotic factors while inhibiting the expression of pro-apoptotic factors, increased the proliferation activity of hypoxic cells, and thereby increased the ability of vascular endothelium cells to resist hypoxic damage [70]. Danggui Buxue decoction is from Li Dongyuan's “Internal and External Injury Distinguishing Theory.” It is composed of astragalus and Chinese Angelica. Danggui Buxue decoction can protect the damaged endothelial progenitor cell function and inhibit cell apoptosis after ox-LDL is induced *in vitro*. The serum containing Danggui Buxue decoction can promote endothelial progenitor cell proliferation, migration, adhesion, tubule function, and cell NO secretion; activate PI3K-dependent or related pathways; and upregulate the expression of eNOS mRNA and total Akt and p-Akt proteins [71]. It is suggested that PI3K/Akt pathway can cause Danggui Buxue decoction to regulate the activity of endothelial progenitor cells, improve their functions, and repair damaged vascular endothelium to prevent AS [71] (Table 2).

## 5. Conclusions and Perspectives

The PI3K/Akt pathway is involved in the occurrence and development of vascular physiology and pathology. The drug targeting of the PI3K/Akt pathway has potential applicability in treating ASCVD and its complications. It is worthwhile for us to explore the use of the PI3K/Akt pathway. Therefore, domestic scholars give full play to the advantages of TCM syndrome differentiation and treatment and screen effective medicinal materials and compound prescriptions. Multidisciplinary methods should be adopted to actively find new breakthrough points from the pathogenesis and explain the intervention effects of TCM from various aspects of stabilizing plaques, repairing vascular endothelial cell damage, regulating cell metabolism, preventing platelet aggregation, inhibiting inflammation, and regulating the homeostasis imbalance of vascular function. The prevention and treatment of AS with TCM have important academic value, and certain results have been achieved. However, the pathogenesis of AS is complicated and requires extensive and in-depth research from multiple angles to enrich the knowledge on the anti-atherosclerotic effect of Chinese medicine and guide further translational research and clinical trials.

## Figures and Tables

**Table 1:** The mechanism of action of Chinese herbal extracts on AS through PI3K/Akt signaling pathway.

Name	Mechanism	Model	References
TS IIA AS IV	Inhibit the translocation of NF-*κ*B to the nucleus and the expression of TLR4. Enhance vascular stability through PI3K/Akt and TLR4/NF-*κ*B signals, reduce inflammation and MMP-9 expression, and inhibit unstable plaques.	ApoE^−/–^ mouse	[48]
Breviscapine	Reduce the levels of inflammatory factors of VCAM-1, ICAM-1, IL-6, and TNF-*α*; reduce the blood lipid levels of SD rats; and reduce cell apoptosis. PI3K/Akt signaling pathway activates Nrf2 to protect cells.	SD rat	[55,61]
Gypenoside	Activate the PI3K/Akt/Bad signaling pathway, regulate the expression of aortic cell apoptosis-related proteins, and downregulate mitochondrial deletion and fusion proteins and mitochondrial energy-related proteins.	ApoE^−/–^ mouse	[59]
Gypenoside XVII	Activation of ER*α*-mediated PI3K/Akt pathway, Nrf2/HO-1 up-regulation, and the level of antioxidant enzymes; reduce ox-LDL-induced oxidative damage; and inhibit ox-LDL-mediated HUVEC apoptosis.	HUVECs ApoE^−/–^ mouse	[60]
Salidroside	Improve endothelial function related to increased eNOS activation, activate AMPK, and improve endothelial function by activating mitochondrial-related AMPK/PI3K/Akt/eNOS pathway.	HUVECs ApoE^−/–^ mouse	[16]
Iso	Activate PI3K/Akt signal, upregulate THP-1-derived macrophages HO-1, prevent ox-LDL-induced apoptosis, and inhibit lipid deposition in ox-LDL-induced macrophage apoptosis.	ApoE^–/–^ mouse	[6]
Quercetin	High fructose activates ROS and inactivates the PI3K/Akt signaling pathway, causing apoptosis and inflammation through the Bcl-2/caspase-3 and IKKa/NF-kB signaling pathways, respectively. Quercetin improves and inhibits the progression of ROS AS through PI3K/Akt.	C57BL/6 induced by high fructose	[51]
Emodin	Regulate blood lipids, inhibit the mTOR signal pathway and PI3K/Akt signal activity, stimulate the body to increase autophagy, and increase the metabolism of glycolipids.	ApoE^−/–^ mouse	[53]

**Table 2:** The mechanism of Chinese herbal compound influencing AS through PI3K/Akt signaling pathway.

Name	Mechanism	Model	Reference
Danhong injection	Improve blood lipid level, reduce AS index and plaque area, inhibit TC level, activate the PI3K/Akt insulin signaling pathway to prevent lipid accumulation in macrophages, and improve the prognosis of AS	ApoE^−/–^ mouse	[62]
Qishen capsules	Regulate blood lipids and inhibit the phosphorylation and overactivation of PI3K and Akt protein	ApoE^−/–^ mouse	[65]
Tongxinluo	Upregulate the expression of HIF in vascular endothelial cells through PI3K/Akt/HIF dependent signaling pathways and improve the ability of vascular endothelial cells to resist hypoxia injury	HUVECs	[70]
Buyang Huanwu decoction	Inhibit the expression of PI3K and p-Akt proteins activated by LPS stimulation and inhibit downstream inflammatory factors	Male rabbit	[64]
Danggui Buxue decoction	Regulate the activity of endothelial progenitor cells through PI3K/Ak t pathway, improve their functions, and repair vascular endothelium	Endothelial progenitor cells New Zealand male rabbit	[71]
